# Supplementary figures and images for: Transcriptional Specificity Analysis of Testis and Epididymis Tissues in Donkey
Source: Genes (Basel). 2022 Dec 11;13(12):2339. doi: 10.3390/genes13122339 (PMC9777602; doi:10.3390/genes13122339)

A

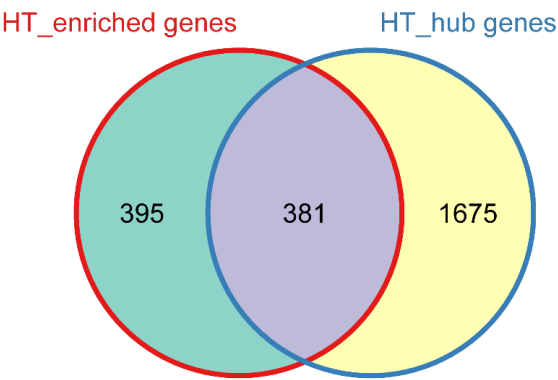

B

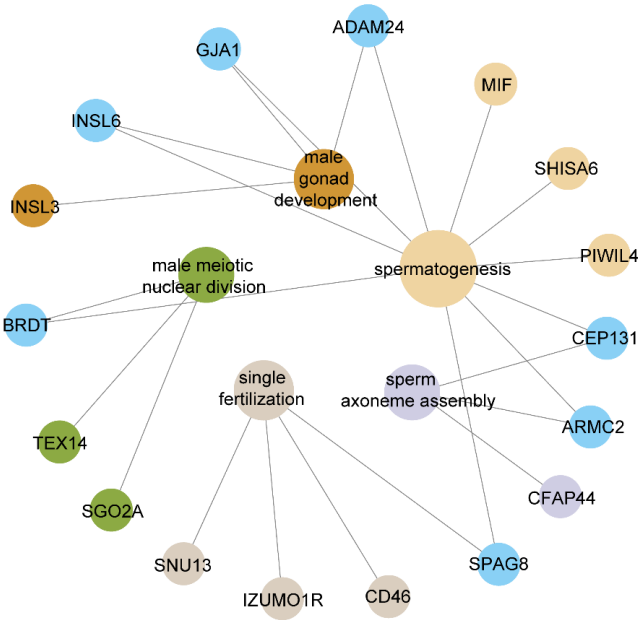

C

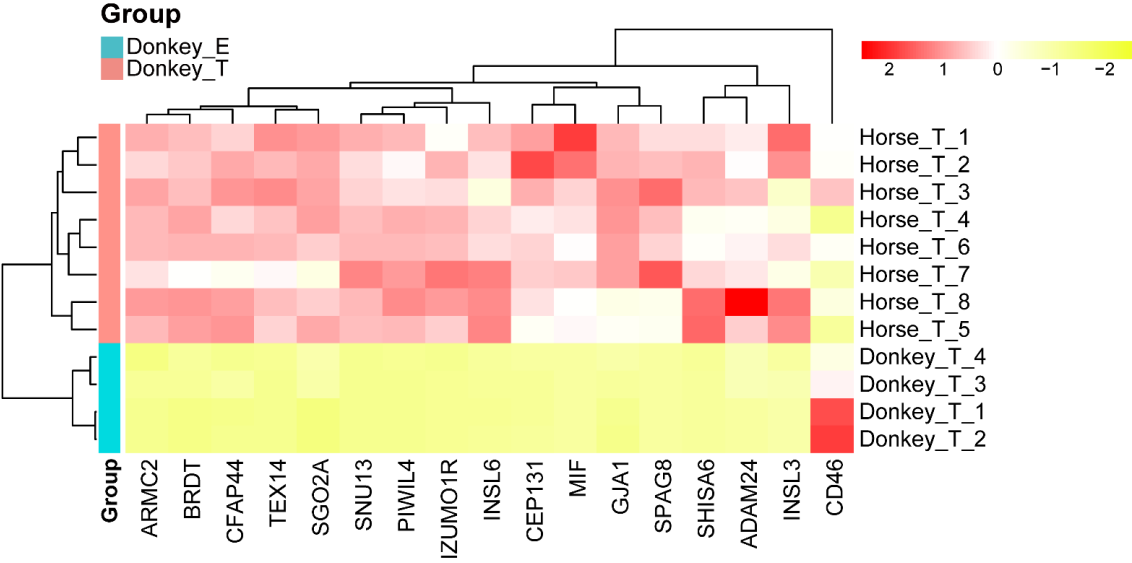

D

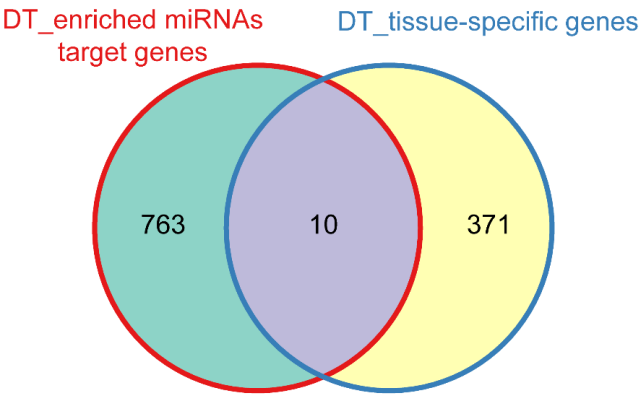

E

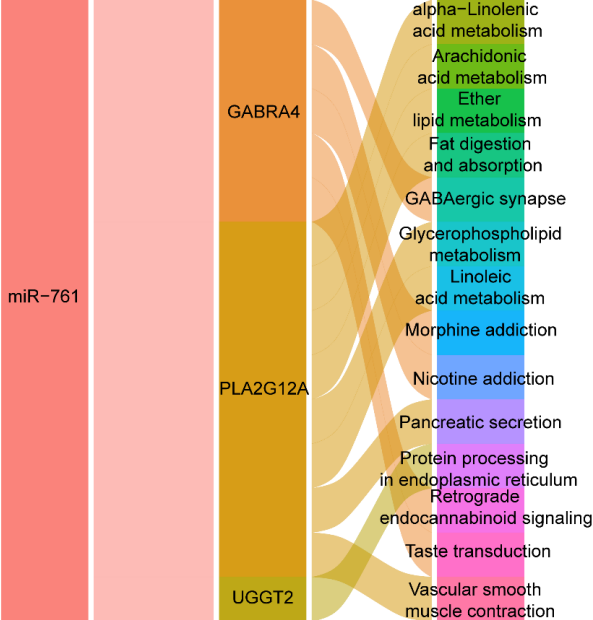

Supplement: Supplementary file 1 [file genes-13-02339-s001.zip › genes-2023081-supplementary/Figure S1.pdf]
